# How Much Will Climate Change Reduce Productivity in a High-Technology Supply Chain? Evidence from Silicon Wafer Manufacturing

**DOI:** 10.1007/s10640-023-00803-4

**Published:** 2023-08-30

**Authors:** Jingnan Chen, Miguel A. Fonseca, Anthony Heyes, Jie Yang, Xiaohui Zhang

**Affiliations:** 1 Economics Department, Business School, University of Exeter, Exeter, EX4 4PU UK; 2 Economics Department, University of Birmingham, Birmingham, B15 2TT UK; 3 Business School, Guilin University of Electronic Technology, Guilin, 541004 Guangxi China; 4 NIPE, Universidade do Minho, Braga, Portugal

**Keywords:** Climate impacts, Adaptation, Productivity

## Abstract

The frequency of hot days in much of the world is increasing. What is the impact of high temperatures on productivity? Can technology-based adaptation mitigate such effects of climate change? We provide some answers to these questions by examining how high outdoor temperatures affect a high-technology, precision manufacturing setting. Exploiting individual-level data on the quantity and quality of work done across 35,190 worker-shifts in a leading NYSE-listed silicon wafer maker in China, we evidence a negative effect of outdoor heat on productivity. The effects are large: in our preferred linear specification, an increase in wet bulb temperature of $10\,^{\circ }$C causes a reduction in output of 8.3%. Temperature effects exist even though the manufacturer’s work-spaces are indoors and protected by high-quality climate control systems. Results are not driven by extreme weather events and are robust to alternative modelling approaches. They illustrate the potential future adverse economic effects of climate change in most of the industrialised world.

## Introduction

One of the many consequences of climate change is the increase in frequency and intensity of hot days. Indeed, most of the highly-populated and highly-industrialised parts of the world (especially in Asia) are expected to face marked increases in the number of hot and very hot days over the next few decades. For example, in rapidly modernising India there were five days with average dry-bulb temperature above $35\,^{\circ }$C ($95\,^{\circ }$F) between 1957 and 2000. But under a business-as-usual scenario, the well-known Hadley model (HadCM3) predicts India will experience 75 such days in a typical year between 2075 and 2100. In the US the same model predicts an increase from 0 to 29 such days over the same period (Burgess et al. 2017). In China, a 1.5$^{\circ }$ rise in global temperature will make extreme warm events twice more likely (Li et al. 2017).

Extreme heat has negative consequences on a variety of human activities. It causes discomfort, fatigue (Nielsen et al. 1993; Galloway and Maughan 1997; González-Alonso et al. 1999) and even cognitive impairment (Epstein et al. 1980; Ramsey 1995; Pilcher et al. 2002; Hancock et al. 2007). Extreme heat restricts the time people can spend in productive activities (Graff-Zivin and Neidell 2014) and has a negative impact on workplace productivity (Zhang et al. 2018; Somanathan et al. 2021), as well as the academic performance of high school students (Goodman et al. 2020; Park 2022). At the macroeconomic level, very high temperatures have a detrimental impact on economic growth (Dell et al. 2012; Burke et al. 2015), as well as on agricultural and industrial output (Dell et al. 2012). Heat could impact human economic performance either directly in the workplace, or indirectly through poor health (Ebi et al. 2017; Degallier et al. 2010), or by undermining one’s recovery capacity through poor rest or sleep (Rifkin et al. 2018), which in turn has a negative impact on economic outcomes (Banks and Dinges 2007; Lim and Dinges 2010; Bessone et al. 2021).

Could the negative impact of extreme heat on productivity be avoided, or at least mitigated, through technological adaptation of our workplaces? Air-conditioning work spaces is most natural, readily-available and effective form of adaptation against extreme heat. Barreca et al. (2016) document that the adoption of residential air conditioning in the 1960 s accounts for most of the decline in the statistical relationship between mortality and extreme heat in the United States. Using data from US high schools, Park et al. (2020) show that heat has a substantial and long-term negative impact on learning, which can be mitigated through air-conditioned classrooms.

We examine the impact of high temperature events on worker productivity in a high-tech industry, whose production technology already incorporates the climate-controlled working environments. In particular, we study data from a leading NYSE-listed Chinese manufacturer of multi-crystalline, 165 $\upmu$m (0.165 mm) silicon wafers, a key component of solar panels. The manufacture of the wafers takes place in a number of workshops at a single main facility in South East China. Employees operate specialised, precision cutting equipment. The delicate nature of the wafers means that, to ensure quality, all workshops have installed climate control systems designed to keep the workshops at a constant temperature of $25\,^{\circ }$C ($77\,^{\circ }$F) and relative humidity level of 60%. We study an archetypal ‘modern economy’ activity. The type of work plausibly parallels that done by many other workers in precision manufacturing settings across the world. The facility that we study is located in a highly-populated and heavily-industrialised part of the world. It experiences many hot days, and their frequency is likely to increase.

We study fine-grained worker productivity data, which includes the number of wafers made in a given shift in a given day, and the quality grade of each wafer. Our estimating sample includes data on 35,190 worker-shift days and relates to 635 separate workers from September 2013 to August 2017. We match this worker productivity data with historical data on local temperature and humidity to estimate the statistical relationship between temperature, humidity and worker productivity. The granularity and coverage of our data means that we observe each worker under many different ‘treatment’ conditions, allowing us to rely on within-worker estimates throughout.

To preview results, taken together our analyses make a persuasive case for there being a substantial and robust negative effect of shift-day temperature on worker productivity, even in the presence of high-tech climate adaptation solutions. In our central linear specification, other things equal, each degree increase in shift-day maximum wet-bulb temperature causes a reduction in productivity of 0.83%. In our binary specification model, a day where maximum wet-bulb temperature exceeds $28\,^{\circ }$C is associated with 5.8% lower productivity compared to a typical day in the estimating sample when it does not.

## Study Setting

The silicon wafer is an important input to twenty-first century living. It is the material from which semiconductors, used in integrated circuits and to be found in all types of electronic devices, are made. It is also the core component of solar cells which are assembled to make photovoltaic (PV) or ‘solar’ panels, used to convert solar energy to electricity.

Manufacturing wafers requires that polycrystalline silicon (polysilicon) be formed into cylindrical ingots which are then sliced very thinly using a multi-wire saw. The process is one of high precision: an average wafer is between 0.16 and 0.24 mms thick.

We obtained privileged access to detailed daily wafer production data from a leading NYSE-listed manufacturer of wafers in China. The company specialises in multicrystalline 165 $\upmu$m (0.165 mm) wafers for supply to the solar industry, and its products are distributed widely within China and exported to many other countries.

The manufacture of wafers takes place in a number of workshops at a single main facility in southeastern China. Employees operate specialized equipment (in particular precision cutting equipment). The delicate nature of the wafers means that, to ensure quality, all workshops have installed climate control systems designed to keep the workshops at a constant temperature of $25\,^{\circ }$C ($77\,^{\circ }$F) and relative humidity level of 60%.

There are a number of wafer workshops in the company manufacturing plant spread across different buildings. Specialized equipment (see Fig. 4a in Appendix) is used in the workshops for wafer manufacturing. The wafer manufacturing process involves slicing a large silicon ingot through densely arranged single-layer wires (see Fig. 4b in Appendix).

The main task for workers in a wafer workshop is to prepare the saw machine, load the ingot onto a precise cutting location, perform the cut, remove the wafers and clean up the machine. Each ingot has a surface area of 158 $\times$ 156 mm and a maximum length of 500 mm. Each ingot can weigh up to 2800 kg (just over 6000 lbs). The resulting wafers are later counted and checked by the quality control department and are each given a grade. The number of wafers are a function of the length of the ingot and the quality of the work performed by the worker.

Attention and care are required from the workers every step of the process. It usually takes several hours to perform one cut. During the cutting, the workers have to perform regular checks on the wire, the water pressure, the electric voltage and the cooling system. They also need to monitor the flow of the slurry. In short, the wafer cutting is a time-consuming, labor intensive task. It is both physically demanding and reasonably sophisticated given the complexity of the machinery.^1^ Variations in output can occur if workers are not careful or attentive. In the time frame of our data set, workshops operated on a day shift from 8:30am to 8:30pm. Workers are expected to perform multiple cuts in a shift.

All workers in our data set were male. The overwhelming majority of workers were experienced: at the earliest date in our data set, 87% of workers had at least one month’s experience with the firm; the median worker had over 9 months’ experience. The majority of workers (66%) operated in the same workshop and shift for the duration of the data set. We have no information as to how workers were allocated to shifts. The remaining workers are recorded working in more than one workshop or shift. Our data set indicates that such workers predominantly worked in a specific workshop (around 90% of the time) and worked in other workshops on other occasions. We assume that workshop/shift changes were likely due to workers covering for an absent colleague, or doing an extra shift.^2^ The overwhelming majority of workers had secondary school education, with a few having completed vocational college-level education.

We analyse detailed data on silicon wafer production from September 2013 to August 2017. Each observation describes an individual cut, including the number of wafers produced from the cut, the type of machine used to perform the cut, the worker who operated the machine, the shift the worker was on, the particular workshop where the cut took place, the cutting time, and the resulting percentages of grade A, A$-$, B, and B$-$ wafers that resulted (the letters denote quality of cut, with A being the best).

A worker executes many cuts per shift; for our preferred outcome productivity metric, we aggregate to a worker-shift count the number of wafers produced. As a robustness check for consistency, we re-estimate our main specification using a worker-shift count of the wafers produced at different grades as the left-hand-side variable.

Our main estimating sample includes data on 35,190 worker-shift days and relates to 635 separate workers. Worker remuneration includes piece-rate and bonus elements, and the temperature treatment effects identified here are understood to be conditional on that pattern of remuneration, and any other idiosyncratic features of our producer.

Our objective is to investigate the causal impact of outdoor temperature in the vicinity of the plant on wafer production at worker-shift level. We take historical weather simulation data on hourly temperature and humidity from www.meteoblue.com, with a spatial resolution at no more than 30 km from the city where the facility is located. The data is complete (no missing points) and we use it in hourly steps and daily aggregations.

As both temperature and humidity are well-established to have human impacts, in this study we adopt wet bulb temperature (WBT) as our central measure of heat (Parsons 2014). WBT combines temperature and relative humidity into a single number that captures the ‘experience’ of heat and is probably the most widely used and validated index for assessing occupational heat stress. It is the measure of heat burden used, for example, by Somanathan et al. (2021). It is approximated by using the following formula (Lemke and Kjellstrom 2012):

1 $$\begin{aligned} WBT=0.567T+0.216\rho +3.38, \end{aligned}$$

where *T* is ambient dry-bulb temperature (in degrees Celsius) and $\rho$ is the water vapor pressure calculated from relative humidity (RH) by:

2 $$\begin{aligned} \rho =\frac{RH}{100}\times 6.105 \exp \left( \frac{17.27T}{237.7+T}\right) \end{aligned}$$

While WBT is our preferred measure of heat, for the purposes of robustness we also present main results using dry-bulb temperature and relative humidity included as separate regressors.

**Fig. 1:**
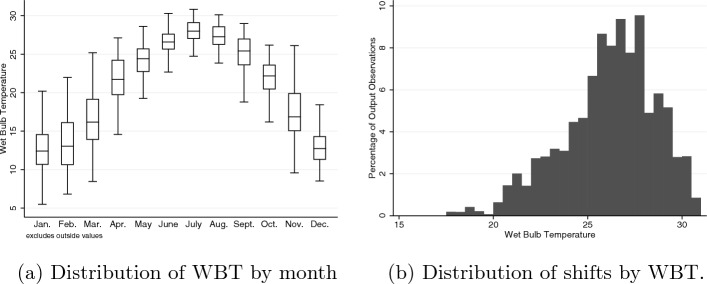
Temperature distributions

Because our focus here is on heat effects, and WBT is typically regarded as valid only at levels above 20, we limit attention to data from the months of May through September inclusive—the hot months of the year. This is consistent with Fig. 1a which shows that almost all days are in the range where WBT is valid and there are few such days outside those months.

**Table 1:** Summary statistics

	Mean	St.d
Output (number of wafers)	9390.37	3892.88
Temperature ($^{\circ }$C)	26.15	2.44
Temperature 24-hr average ($^{\circ }$C)	24.27	2.34
Temperature daytime average ($^{\circ }$C)	24.04	2.31
Temperature ($^{\circ }$C)>24	0.82	0.39
Temperature ($^{\circ }$C)>26	0.57	0.49
Temperature ($^{\circ }$C) >28	0.22	0.42
Precipitation (mm)	5.73	13.21
Cloud coverage (%)	59.49	33.76
Sunshine duration (min)	256.94	246.17
Wind gust (km/h)	10.02	5.63
Wind speed (km/h)	7.52	3.73
Observations	35,190	
Workers	635	

Table 1 presents summary statistics for variables used in the main analysis.^3^ Figure 1b shows that there is a substantial variation in the preferred temperature metric (maximum daily WBT) across shifts. It is this variation on which identification is based.

## Empirical Strategy

We estimate a series of worker fixed-effects models that incorporate temperature in several different ways. Our base specification is:

3 $$\begin{aligned} Output_{i,t}=\alpha _{0}+f(Temp(t))+X_{t}\alpha +u_{i}+v_{year}+w_{month}+\epsilon _{i,t}, \end{aligned}$$

$$Output_{i,t}$$ is the production (count of wafers) by worker *i* on the day-shift on day *t*. $$Temp_{t}$$ indicates WBT on date *t*, *Temp*(*t*) is the vector of temperature measures and *f*(.) is a parametric function. In the linear specification $$f(Temp(t))=\beta _{1}Temp_{t}$$. The vector $$X_{t}$$ contains other date-level meteorological variables. The terms $$u_{i}$$, $$ v_{year}$$ and $$w_{month}$$ are worker, year and month fixed effects (FEs) respectively.

*Non-linear models* A challenge in exploring heat effects is the potential for non-linearity and we address this in three ways. First, consistent with common empirical practice we estimate a quadratic specification in which $f(Temp(t))=\beta _{1}Temp_{t}+\beta _{2}Temp_{t}^{2}$. Second, we set $f(Temp(t))=\beta _{1}I(Temp_{t}>T_{high})$ where $I(\cdot )$ is an indicator function and $T_{high}$ is some threshold value. Depending on the threshold, $T_{high}$ is variously set equal to $24\,^{\circ }$C, $26\,^{\circ }$C and $28\,^{\circ }$C. Finally, we consider a semi-parametric model in which we use a set of dummy variables corresponding to $3\,^{\circ }$C temperature bins. The width of the bins was selected to ensure roughly equal numbers across bins. This model will capture any non-linear effects without assuming a functional form.

*Inter-day effects* Besides examining the impact of same-day temperature on workers productivity we also investigate possible lagged and inter-day effects. The potential for complex lagged relationships and existence of serial correlation in the treatment variable makes pinning down any exact dynamic structure empirically challenging, and we approach this in several different ways.

First, defining

$$\begin{aligned} f(Temp(t))=\beta _{1}\times \frac{Temp_{t}+Temp_{t-1}+Temp_{t-2}}{3}, \end{aligned}$$

we estimate the impact of the mean daily maximum WBT over a three-day period on output in day *t* (and analogous five-day and seven-day periods).

Second, we report a weekly analysis which regresses worker-level output over a five day working week with mean daily maximum WBT during that working week.

Third, we estimate lag structures by applying finite distributed-lag models. In particular to apply a finite distributed lag model with *s* lags would imply setting *f*(*Temp*(*t*)) as

4 $$\begin{aligned} f(Temp(t))=\beta _{0}Temp_{t}+\beta _{1}Temp_{t-1}+... +\beta _{s}Temp_{t-s}. \end{aligned}$$

The coefficients from this model allow for estimation of the impact on productivity of both temporary (single-day) and longer term changes in temperature.

Auto-correlation in daily maximum WBT means that the specification in equation (4) suffers from multicollinearity, and to mitigate this we also apply polynomial distributed lag models (Parsons 2014). Such models constrain the admissible form of lagged effects by restricting attention to a coefficient sequences that satisfy a low degree polynomial; we report results from the quadratic model in the main text and quartic polynomials as a robustness check.

The quadratic and quartic models generate coefficients on same-day and lagged values of temperature that best fit the data subject to the lag structure taking, respectively, the form

5 $$\begin{aligned} \beta _{j}=\eta _{0}+\eta _{1}j+\eta _{2}j^{2},\;\;\;j=0,1,...,s. \end{aligned}$$

and

6 $$\begin{aligned} \beta _{j}=\gamma _{0}+\gamma _{1}j+\gamma _{2}j^{2} +\gamma _{3}j^{3}+\gamma _{4}j^{4},\;\;\;j=0,1,...,s. \end{aligned}$$

The steps required to execute these specifications are reported in the Appendix.

## Results

**Fig. 2:**
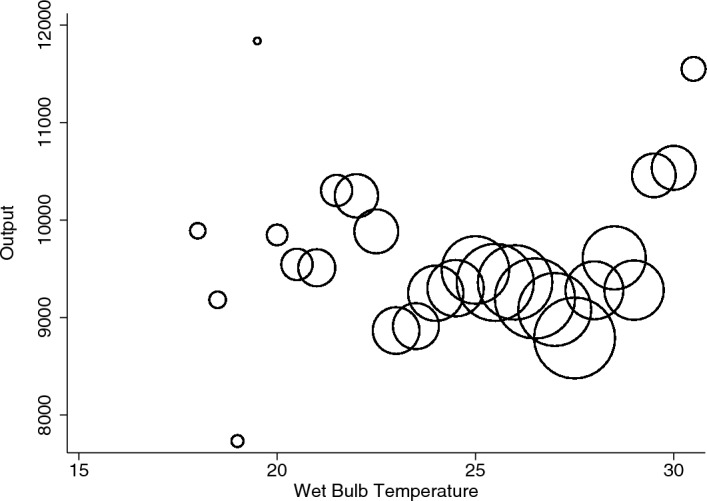
Temperature and output

Before reporting our main results, we present a simple data plot. Figure 2 plots residual worker-shift output from a regression containing only worker, month and year fixed effects against shift-day temperature. These are binned and the size of markers in the figure is proportional to the number in each bin.

While we can see a few outliers (particularly at the right-hand end), most observations fall in the 22–$28\,^{\circ }$C range. The plot suggests a negative relationship between temperature and output. It also clarifies that any negative effects that the regression analysis that follows will generate are not being driven by extreme heat realisations, but rather by impacts across a wider interval of mid-range realisations. We will confirm this intuition by conducting a number of different outlier exercises.

### Contemporaneous Effect of Heat

Table 2 shows the results from a model estimating a linear relationship between WBT and worker-shift output. Our preferred specification includes worker, month, and year fixed effects, as well as weather controls. The estimated slope coefficient in our preferred specification is $-$78.00, which implies that a $1\,^{\circ }$C increase in WBT causes a reduction of 78 wafers produced by a worker in a shift. Across the whole sample, the average number of wafers produced by a worker per shift is 9390, so this constitutes an.83% fall in output per 1-degree increase. The estimates are similar in magnitude to the most saturated model (5); the coefficient is slightly smaller in most other specifications, but still implying an economically important effect: the smallest estimated effect (Column 2) amounts to a.54% fall in output per 1-degree increase. For completeness, we include a specification (6) with Year $\times$ Month fixed effects, which capture any changes over time in the seasonality of temperature and productivity. That is, this specification controls for the fact that summers might be getting hotter over time, and that may drive any results. The fact that we only have one geographical location may hinder the effectiveness of this approach, since we may not have enough variation in temperature, which may lead to imprecise estimates. Figures 6 and 7 in the Appendix plot monthly average temperature and monthly average productivity over time, respectively. We cannot find major changes in seasonality from the plot over the time series, which suggests that that changes in seasonality over time are not driving the results. Furthermore, Fig. 8 in the Appendix plots the residuals of the regression of WBT on year-by-month fixed effects. Other than the case of $29\,^{\circ }$C, the relationship is, in the main, flat around zero. Residuals are negative below $20\,^{\circ }$C, but they are a very small proportion of the sample. We conclude that indeed there is lack of variation in the data once we control for year-by-month fixed effects, which in turn contributes to the lack of precision of the estimates in that regression.

**Table 2:** Estimates from linear model

	(1)	(2)	(3)	(4)	(5)	(6)	(7) Pref
WBT	$$-$$57.941$$^{***}$$	$$-$$51.245$$^{***}$$	$$-$$56.614$$^{***}$$	$$-$$55.967$$^{***}$$	$$-$$76.030$$^{***}$$	$$-$$8.857	$$-$$78.001$$^{***}$$
	(9.170)	(10.433)	(9.156)	(9.113)	(9.901)	(8.880)	(9.869)
Precipitation					$$-$$6.225$$^{***}$$	$$-$$3.325$$^{***}$$	$$-$$6.348$$^{***}$$
					(1.165)	(1.166)	(1.165)
Cloud cover					$-$0.984	0.614	$-$0.996
					(1.142)	(1.119)	(1.131)
Sunshine duration					0.114	0.169	0.104
					(0.155)	(0.145)	(0.154)
Wind gust					$$-$$16.144$$^{***}$$	$$-$$3.220	$$-$$16.849$$^{***}$$
					(5.528)	(5.030)	(5.386)
Wind speed					12.210$$^{***}$$	$$-$$8.444	14.721$$^{***}$$
					(7.773)	(7.139)	(7.654)
Obs	35,190	35,190	35,190	35,190	35,190	35,190	35,190
No. of workers	635	635	635	635	635	635	635
Worker FE	Yes	Yes	Yes	Yes	Yes	Yes	Yes
Year $\times$ Month FE	No	No	No	No	No	Yes	No
Year FE	Yes	Yes	Yes	Yes	Yes	No	Yes
Month FE	Yes	No	Yes	Yes	Yes	No	Yes
Week FE	No	Yes	No	No	No	No	No
Day of week	No	No	Yes	Yes	Yes	No	No
Building FE	No	No	No	Yes	Yes	No	No

The dependent variable in all regressions is output per worker-shift. WBT refers to the maximum wet bulb temperature on day of shift. Robust standard errors clustered at the worker level are presented in parentheses, and ***$p<0.01$, **$p<0.05$, *$p<0.1$. The sample comprises all day shifts from May to September

Existing research has identified non-linear relationships between temperature and a range of human outcomes. Indeed, the relationship between temperature and worker productivity could not just be non-linear but in fact non-monotonic: increasing up to some ‘comfortable’ mid-range value and decreasing thereafter. Focusing only on summer months plausibly mitigates the worst of these concerns – restricting attention to days on the hotter-than-comfortable part of the support - but not necessarily completely. As such we consider a number of alternative, non-linear, models.

Columns 1–6 in Table 3 display the results from a model estimating a quadratic relationship between WBT and output under different specifications. In our preferred specification (column 6) output has an inverted-u relationship as a function of WBT: output rises at low temperatures, reaching an estimated ‘turning point’ at $23.3\,^{\circ }$C, with reasonably tight bounds (95% confidence interval: [$22.6\,^{\circ }$C, $23.9\,^{\circ }$C]). The estimates are robust to the inclusion of building-specific fixed effects and other weather controls.

Another alternative non-linear specification involves defining an indicator variable that equals 1 if the maximum temperature on day *t* exceeds some threshold and zero otherwise. In other words, we estimate the independent effect of a day being ‘hot’ compared to ‘non-hot’. Columns 7–9 display the results using $24\,^{\circ }$C, $26\,^{\circ }$C, and a $28\,^{\circ }$C thresholds. Temperatures exceeding $28\,^{\circ }$C reduce the number of wafers produced by a worker in a shift by 548 or 5.8% compared to a reference day in which that threshold is not exceeded. Again, this is a substantial effect.

Finally, we estimate a semi-parametric model, in which we do not impose any functional form to the relationship between WBT and output. We achieve this by regressing output on a set of dummies for $3\,^{\circ }$C temperature bins (to ensure equal numbers across bins; the omitted category is WBT $\le 21\,^{\circ }$C). Column 10 displays the estimates. The model hints at a quadratic relationship: there is an initial non-significant increase in productivity when WBT rises from <21$\,^{\circ }$C to 21–24$\,^{\circ }$C, followed by a decline thereafter, which becomes very marked at high temperatures. Again, this points to our parametric estimates being very robust, without rejecting outright the linear model (from which we derive our headline result).

**Table 3:** Estimates from non-linear models

	(1)	(2)	(3)	(4)	(5)	(6)	(7)	(8)	(9)	(10)
	Quadratic						Binary			Semi-parametric
WBT	808.544***	410.148***	838.167***	871.742***	1031.185***	976.773***				
	(123.181)	(126.537)	(123.641)	(124.821)	(131.367)	(129.026)				
WBT$^2$	$-$17.097***	$-$9.124***	$-$17.654***	$-$18.304***	$-$22.049***	$-$21.006				
	(2.418)	(2.500)	(2.429)	(2.454)	(2.603)	(2.551)				
Precipitation					$-$5.556***	$-$5.798***				
					(1.156)	(1.154)				
Cloud cover					$-$1.756	$-$1.715				
					(1.167)	(1.150)				
Sunshine duration					0.195	0.183				
					(0.156)	(0.156)				
Wind gust					$-$18.833***	$-$19.658***				
					(5.584)	(5.423)				
Wind speed					18.034***	20.679***				
					(7.789)	(7.652)				
WBT > 24							$-$202.452***			
							(43.268)			
WBT > 26								$-$213.827***		
								(45.753)		
WBT > 28									$-$547.701***	
									(52.815)	
Turning point	23.6 $^{\circ }$C	22.5 $^{\circ }$C	23.7 $^{\circ }$C	23.8 $^{\circ }$C	23.4 $^{\circ }$C	23.3 $^{\circ }$C				
	[22.9 $^{\circ }$C, 24.4 $^{\circ }$C]	[20.6 $^{\circ }$C, 24.3 $^{\circ }$C]	[23.1 $^{\circ }$C, 24.4 $^{\circ }$C]	[23.2 $^{\circ }$C, 24.4 $^{\circ }$C]	[22.8 $^{\circ }$C, 24.0 $^{\circ }$C]	[22.6 $^{\circ }$C, 23.9 $^{\circ }$C]				
$21<\text {WBT}\le 24$										119.190
										(107.797)
$24<\text {WBT}\le 27$										$-$21.735
										(108.668)
$27<\text {WBT}\le 30$										$-$321.318***
										(113.384)
$30<\text {WBT}$										$-$478.572***
										(156.643)
Observations	35,190	35,190	35,190	35,190	35,190	35,190	35,190	35,190	35,190	
Number of works	635	635	635	635	635	635	635	635	635	635
Worker FE	Yes	Yes	Yes	Yes	Yes	Yes	Yes	Yes	Yes	Yes
Year FE	Yes	Yes	Yes	Yes	Yes	Yes	Yes	Yes	Yes	Yes
Month FE	Yes	No	Yes	Yes	Yes	Yes	Yes	Yes	Yes	Yes
Week FE	No	Yes	No	No	No	No	No	No	No	No
Day of Week	No	No	Yes	Yes	Yes	No	No	No	No	No
Building FE	No	No	No	Yes	Yes	No	No	No	No	No
Other weather	No	No	No	No	Yes	Yes	Yes	Yes	Yes	Yes

The dependent variable in all regressions is output per worker-shift. WBT refers to the maximum wet bulb temperature on day of shift. Robust standard errors clustered at the worker level are presented in parentheses, and ***$p<0.01$, **$p<0.05$, *$p<0.1$. 95% confidence intervals of the turning point of the quadratic relationship between wet bulb temperature and output are presented in square brackets. The sample comprises all day shifts from May to September

### Inter-day Effects

So far we have limited our analysis to the contemporaneous effect of temperature on productivity. However, high temperatures could have a long-lasting or even cumulative impact on productivity.

Table 4 shows results from estimates of a regression of worker-shift output on the average maximum WBT over the previous 3, 5, and 7 days, respectively. The effects are again sizeable: an increase in mean daily maximum WBT of $1\,^{\circ }$C over the last 3 days leads to an average decrease in output of 141 wafers (1.5%). Over 5 days, the effect is larger ($-$179, 1.9%), and the effect over 7 days is larger still: 221 fewer wafers or a decline of 2.4%.

**Table 4:** Inter-day effects

	(1)	(2)	(3)	(4)
	Daily	Daily	Daily	Daily average
Average WBT last 3 days	$-$141.168***			
	(12.359)			
Average WBT last 5 days		$-$178.997***		
		(14.765)		
Average WBT last 7 days			$-$221.540***	
			(17.134)	
Average WBT in week				$-$97.857***
				(14.427)
Observations	35,190	35,190	35,190	12,023
Number of workers	635	635	635	446
Worker FE	Yes	Yes	Yes	Yes
Year FE	Yes	Yes	Yes	Yes
Month FE	Yes	Yes	Yes	Yes
Other weather	Yes	Yes	Yes	Yes

The dependent variable in regressions (1)–(3) is output per worker-shift. WBT refers to the maximum wet bulb temperature on day of shift. The dependent variable in regressions (4) is average daily output per worker-shift during a week. Average WBT refers to mean daily maximum WBT. Robust standard errors clustered at the worker level are presented in parentheses, and ***$p<0.01$, **$p<0.05$, *$p<0.1$. The sample of specification (1)–(3) comprises all day shifts from May to September. The sample of specification (4) comprises average of day shifts by workers worked 5 or 6 days over a week in May to September

### Cumulative Effect of Heat

It is plausible to assume that the effects of heat do not all occur simultaneously, but are distributed over time. That is, a particular hot day can influence outcomes not just that day but also on the next day, the day after, and so on.^4^ PDL models are used when collinearity is expected to be a serious problem, as in our setting. They involve imposing a time horizon (the number of lags to consider) and restricting attention to estimates of the weights on lag terms that satisfy a particular polynomial structure (Almon 1965), since it is plausible that the more recent experiences of temperature may be more impactful than those in the more distant past.

We follow common practice in the application of PDLs by imposing a quadratic structure on the lag weights—we report results for a quartic function for purposes of robustness. We estimate models with 5 and 10 lags, corresponding to different time horizons.

Though it is reasonable from first principles (as well as consistent with the results in Table 4) to assume that the effect of high temperature decays over time –in other words productivity on day *t* would be more sensitive to temperature on day $(t-1)$ than on day $(t-2)$, and so on—the restriction that the rate of decay be quadratic in pattern is *ad hoc*. However, in each case we report a specification test that fails to reject the null hypothesis that the pattern observed in the data is consistent with the PDL model versus the corresponding unrestricted finite distributed lag model.

PDL models separately generate (a) estimates of same day impact and (b) estimates of the cumulative impact, summed over the specified number of periods over which the model is estimated.

Table 5 displays the estimation results. The estimated same-day effect of a $1\,^{\circ }$C increase in temperature is rather stable across models, with an implied reduction in productivity per worker-shift of between 22.6 and 33.4 wafers. The estimated cumulative effect of a $1\,^{\circ }$C increase in maximum WBT on the shift day as well as each of the preceding five days is a reduction in productivity of 79.9 wafers per worker-shift. The corresponding cumulative effect of a $1\,^{\circ }$C temperature increase over 10 days is to reduce the productivity by 61.9 wafers per worker-shift. Estimates from the quartic model for the contemporaneous effect are similar to those of the quadratic model; however, the estimated cumulative effect is substantially higher in the quartic model, regardless of the number of lags.

**Table 5:** Polynomial distributed lag model

Polynomial	Quadratic		Quartic	
Number of lags	5	10	5	10
Impact effect (same day)	$-$33.405***	$-$22.260***	$-$32.660***	$-$29.892***
	(8.461)	(6.058)	(10.384)	(9.056)
Cumulative effect	$-$79.886***	$-$61.903***	$-$138.392***	$-$97.132***
	(11.653)	(11.391)	(14.319)	(13.018)
Polynomial specification test				
test-stat ($\chi (1)$)	0.68	2.10	0.88	1.58
*P*-value	0.409	0.147	0.349	0.208
Observations	35,190	35,190	35,190	35,190
Number of workers	635	635	635	635
Worker FE	Yes	Yes	Yes	Yes
Year FE	Yes	Yes	Yes	Yes
Month FE	Yes	Yes	Yes	Yes
Other weather	Yes	Yes	Yes	Yes

Robust standard errors clustered at the worker level are presented in parentheses, and ***$p<0.01$, **$p<0.05$, *$p<0.1$. *P*-values of the polynomial specification tests indicate none of the polynomial distributed lagged model specifications can be rejected. The sample comprises all day shifts from May to September

### Robustness

We have presented results from a number of different approaches, and for most of these, we estimate a number of alternative specifications. Here we report additional robustness tests and falsification exercises applied to the linear model.

**Table 6:** Robustness checks

	(1)	(2)	(3)	(4)	(5)	(6)
	Preferred	All months Temp. > 20	Temperature daytime average	Temperature 24-hr average	Pollution controls	Worker factor controls
WBT	$-$78.001***	$-$80.435***			$-$107.325***	$-$75.706***
	(9.869)	(8.942)			(13.992)	(10.953)
WBT				$-$68.001***		
24-hr average				(9.024)		
WBT			$-$62.814***			
daytime average			(9.135)			
Observations	35,190	53,455	35,190	35,190	24,546	29,949
Number of workers	635	786	635	635	437	331
Worker FE	Yes	Yes	Yes	Yes	Yes	NO
Year FE	Yes	Yes	Yes	Yes	Yes	Yes
Month FE	Yes	Yes	Yes	Yes	Yes	Yes
Other weather	Yes	Yes	Yes	Yes	Yes	Yes

The dependent variable of all regressions is output per worker-shift. WBT refers to the maximum wet bulb temperature on day of shift. Temperature daytime average refers to the average wet bulb temperature from 8am to 8pm on the shift day. Temperature 24-hr average refers to the average wet bulb temperature over 24 h on the shift day. Robust standard errors clustered at the worker level are presented in parentheses, and ***$p<0.01$, **$p<0.05$, *$p<0.1$

*Alternative estimating sample* In defining the sample we were explicit that (a) the research question relates to the effect of heat (not cold) on productivity and (b) the best measure of heat stress is WBT, which is typically regarded as valid only above $20\,^{\circ }$C. Recognising this, and in light of the patterns of temperature summarised in Fig. 1a, led us to restrict the sample to the calendar months of May through September. An alternative approach would have been to take shifts from all months of the year in days where WBT exceeded $20\,^{\circ }$C. This increases the sample size by 18,265. The results of re-estimating the preferred linear specification on this alternative sample are similar to those derived from the restricted sample (see Table 6).

*Alternative temperature measures* We used daily maximum WBT as our preferred measure of temperature. WBT combines dry-bulb temperature and humidity. We estimate a model in which dry-bulb temperature and humidity enter as separate regressors (see Table 11 in Appendix). We also estimated our model with WBT as the regressor, but taking the daytime (8 am–8 pm) mean WBT and the 24-hour (midnight - midnight) mean WBT respectively (see Table 6). Because the regressor definition has changed, the coefficients are not directly comparable, but remain consistent with earlier results (Table 7).

**Table 7:** Estimates from linear model using dry bulb temperature

	(1)	(2)	(3)	(4)	(5)	(6)
Dry Bulb Temperature	$-$32.965***	$-$27.726***	$-$31.334***	$-$33.113***	$-$57.412***	$-$259.628***
(DBT)	(6.329)	(6.947)	(6.341)	(6.311)	(7.324)	(75.035)
Relative Humidity	$-$6.816**	$-$12.032***	$-$5.665**	$-$8.448***	5.306*	$-$64.527**
(RH)	(2.754)	(2.790)	(2.798)	(2.706)	(3.150)	(25.289)
DBT X RH						2.200***
						(0.795)
Observations	35,190	35,190	35,190	35,190	35,190	35,190
Number of workers	635	635	635	635	635	635
Worker FE	Yes	Yes	Yes	Yes	Yes	Yes
Year FE	Yes	Yes	Yes	Yes	Yes	Yes
Month FE	Yes	No	Yes	Yes	Yes	Yes
Week FE	No	Yes	No	No	No	No
Day of week	No	No	Yes	No	No	No
Building FE	No	No	No	Yes	No	No
Other weather	No	No	No	No	Yes	Yes

The dependent variable in all regressions is output per worker-shift

Robust standard errors clustered at the worker level are presented in parentheses, and ***$p<0.01$, **$p<0.05$, *$p<0.1$

The sample comprises all day shifts from May to September

*Alternative productivity measure* Every wafer produced by a worker on a shift is quality-rated and we observe both total product and the amount produced at specific quality grades, from A (highest) to B- (lowest). Table 8 replicates our preferred linear model specification using as the dependent variable total output (model 1), as well as each individual grade models (2-5).

We find a significant negative coefficient on WBT in all grades except B-, where the coefficient on WBT is positive and significant. In other words, the evidence suggests that WBT has a detrimental impact on output quality, as opposed to on output – which would result if the firm would lower production on extremely hot days in order to offset the cost of maintaining the workshops at 21 $^{\circ }$C.

**Table 8:** Output quality

	(1)	(2)	(3)	(4)	(5)
	Total	Grade A	Grade A-	Grade B	Grade B-
WBT	$-$78.001***	$-$68.120***	$-$3.218***	$-$9.878***	2.192**
	(9.869)	(8.609)	(1.156)	(1.829)	(0.938)
Other weather	Yes	Yes	Yes	Yes	Yes
Obs	35,190	35,190	35,190	35,190	35,190
No. of workers	635	635	635	635	635

*Pollution controls* An important recent literature points to air quality having a causal effect on worker productivity, even among those working in indoor environments (Chang et al. 2019; Heyes et al. 2019). To verify this further, we augmented out model with daily pollution controls. These comprised daily average measures of PM2.5, PM10, SO$_{2}$, NO$_{2}$, CO, and O$_{3}$, measured for the city where the plant is located (Table 6).^5^ Results are not disturbed qualitatively and the implied temperature effect is, if anything, somewhat larger.

*Worker random effects* Our results are qualitatively unchanged if we control for worker characteristics (age, years in education, and the log-transformed measure of worker experience at the facility in days) and estimate our model by worker random effects rather than worker fixed effects.

*Night-time temperature* One of the possible mechanisms through which temperature may affect productivity is by negative physical recovery and fatigue. To investigate this channel, we incorporated the previous night’s WBT as an additional regressor (in both linear and quadratic forms) in our linear and quadratic day time temperature models. We find significant coefficients on night-time WBT suggesting that it may have an important effect on productivity. Humid heat increases wakefulness, decreases rapid eye movement sleep and slow-wave sleep; it also suppresses the natural decrease in core body temperature that occurs during night-time sleep. Humid heat may also increase heat stress because the additional air humidity prevents the body’s natural sweat response, since the skin remains wet (Okamoto-Mizuno and Mizuno 2012). Sleep, in turn, is positively correlated with productivity (Gibson and Shrader 2018). This evidence suggests that heat impairs workers’ ability to recover and through it, productivity.

While night-time WBT seems to have an important role in determining productivity, the sign and significance of the coefficients on contemporaneous day-time temperature effect are unchanged, though somewhat smaller in magnitude. However, the fact that our same-day effect remains suggests other mechanisms are also responsible for lower productivity (Table 9).

**Table 9:** Lagged night-time WBT effects

	(1)	(2)	(3)	(4)
Day WBT	$-$55.943***	$-$50.935***	851.939***	764.446***
	(10.079)	(10.164)	(130.440)	(129.152)
Day WBT$^{2}$			$-$18.163***	$-$16.332***
			(2.588)	(2.562)
Night WBT	$-$69.754***	387.761***	$-$56.658***	273.542***
	(9.105)	(93.082)	(9.206)	(92.446)
Night WBT$^{2}$		$-$10.284***		$-$7.451***
		(2.062)		(2.047)
Observations	35,190	35,190	35,190	35,190
Number of workers	635	635	635	635
Worker FE	Yes	Yes	Yes	Yes
Year FE	Yes	Yes	Yes	Yes
Month FE	Yes	Yes	Yes	Yes
Week FE	No	No	No	No
Day of Week	No	No	No	No
Building FE	No	No	No	No
Other weather	Yes	Yes	Yes	Yes

The dependent variable of all regressions is output per worker-shift. Day WBT refers to the maximum wet bulb temperature on day of shift. Night WBT refers to the minimum wet bulb temperature of the last night of the day shift. Robust standard errors clustered at the worker level are presented in parentheses, and ***$p<0.01$, **$p<0.05$, *$p<0.1$. The sample comprises all day shifts from May to September

*Outliers* To verify that the results are not being driven by a small set of extreme values, we executed a number of exercises to remove or reduce the effect of outliers on estimates (see Table 10). First, we remove (column 1) and winsorise (column 2) the top and bottom 5% of shifts by temperature. Second, we remove (column 3) and winsorise (column 4) the top and bottom 5% of worker-shifts by productivity. Our qualitative conclusions remain unchanged in each case.

**Table 10:** Outliers

	(1)	(2)	(3)	(4)
	Trim WBT	Winsorize WBT	Trim output	Winsorize output
WBT	$-$105.599***	$-$98.335***	$-$67.055***	$-$71.912***
	(11.558)	(10.296)	(8.456)	(8.766)
Observations	31,765	35,190	31,667	35,190
Number of workers	589	635	536	635
Worker FE	Yes	Yes	Yes	Yes
Year FE	Yes	Yes	Yes	Yes
Month FE	Yes	Yes	Yes	Yes
Other weather	Yes	Yes	Yes	Yes

The dependent variable of all regressions is output per worker-shift. WBT refers to the maximum wet bulb temperature on day of shift. Trim temperature (output) excludes observations with temperature (output) higher than the 95th percentile and lower than the 5th percentile. Winsorize temperature (output) indicates that temperature (output) below the 5th percentile set to the 5th percentile, and above the 95th percentile set to the 95th percentile. Robust standard errors clustered at the worker level are presented in parentheses, and ***$p<0.01$, **$p<0.05$, *$p<0.1$

*Placebos* Placebo tests are tests of study design. If estimating the chosen specification, but replacing the true value of the regressor of interest with an alternative we know should be irrelevant, delivers significant results then we know those to be spurious and therefore generated by a flaw in the study design.

We conduct a ‘meta’-placebo exercise based on repeated within-sample randomization. First, true WBT on date of shift was replaced by temperature from another, randomly-chosen date in sample without replacement (if a date was assigned a placebo temperature from another date in the same month of the same year then the observation was dropped). Second, the specification from column 6 of Table 3 was estimated using the resulting placebo temperature series and the resulting coefficient and t-statistic on the temperature variable collected.

**Fig. 3:**
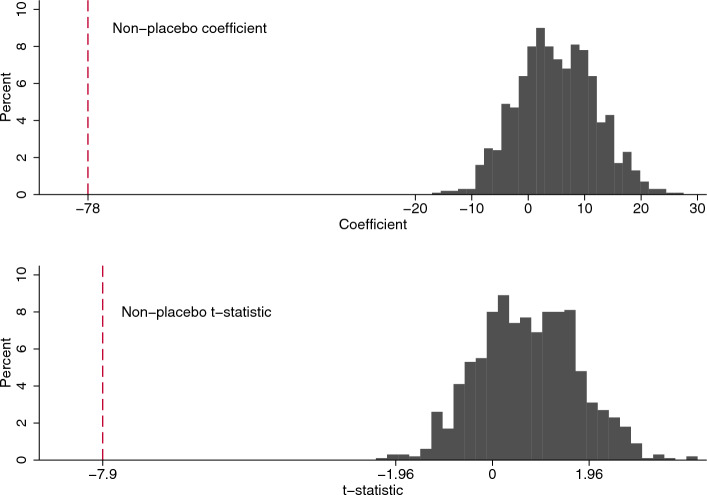
Placebo

**Fig. 4:**
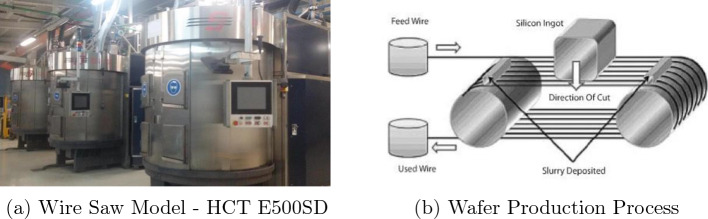
Solar wafers production equipment and process

**Fig. 6:**
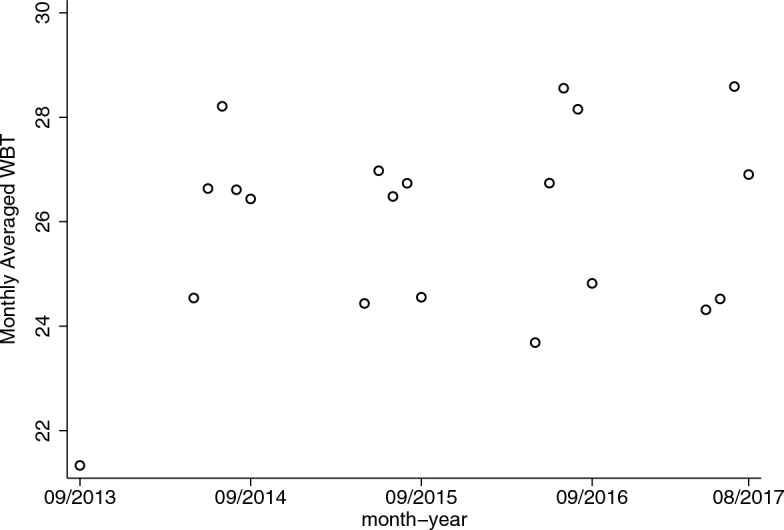
WBT over time

**Fig. 7:**
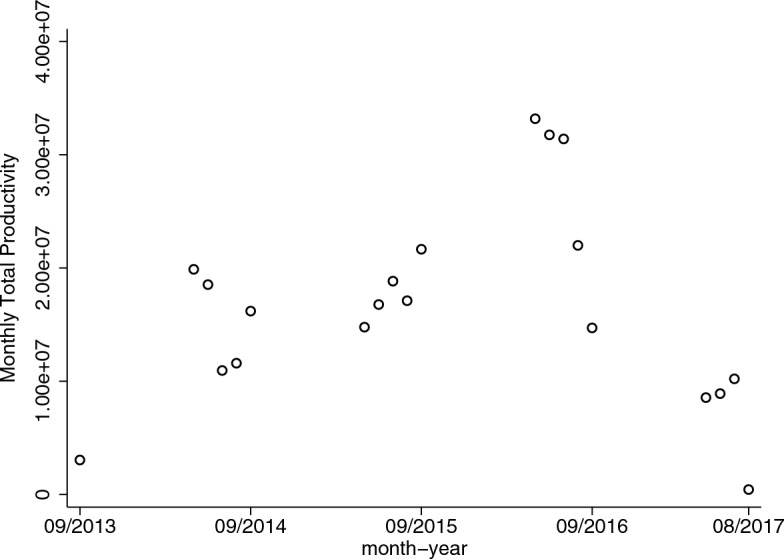
Productivity over time

**Fig. 8:**
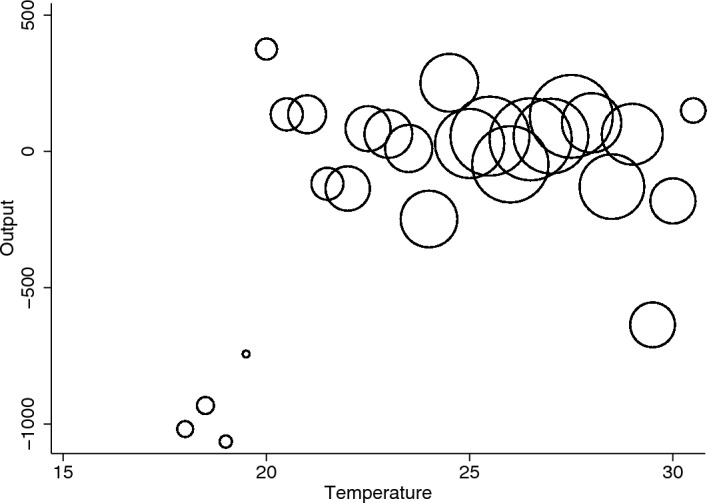
Residuals of regression with year-by-month FEs

This process was repeated with 1000 randomizations. The bar charts in Fig. 3 summarise the coefficients and t-statistics harvested. There is some variation, as would be expected. The lack of symmetry around zero suggests, if anything, something in the study design that imparts an *upward* bias on the temperature coefficient. None of the placebo runs generate values anywhere close to those derived under true assignment, denoted by the dashed vertical lines, which reinforces our confidence in the results.

*Absenteeism* Extreme heat may cause workers to miss work. Absenteeism can lead to lower productivity because it forces the firm to replace workers across shifts (leading to potentially less rest time), or by employing casual workers, which could have a detrimental effect on the productivity of experienced workers. We could not access company HR records about planned or unplanned shift changes. However, we calculated the number of workers working on a given shift. We regressed that variable on WBT as well as month and year FEs, as well as a specification including year, week and day-of-week FEs. Table 15 in the Appendix summarizes the results. We find that a 1 degree increase in WBT leads to a reduction by one worker in shift attendance.

## Discussion and Conclusion

Climate change is likely to result in higher average temperature and more extremely hot days. However, the impact that will have on economic outcomes remains disputed (Stern 2007). While economy-level models have been prominent in debate in recent years, evidence of the mechanisms linking outdoor temperature to key economic drivers at micro-level is inadequately developed. The results here contribute to the nascent literature of the causal impact of outdoor temperature on skilled labour productivity, complementing important recent evidence (Somanathan et al. 2021).

In addition to studying a new setting, we also take forward the challenge of unpicking possibly non-linear and lagged relationships of temperature on productivity, applying a range of tools. Our results provide compelling evidence that high outdoor temperatures have a substantial negative effect on the productivity of skilled labour, even when production takes place in climate controlled spaces.

The fine-grained nature of the data allows for within-worker estimation, netting out a wide set of time-invariant but unobservable individual characteristics that would confound results from cross-sectional analysis. The effects we find are large: in our preferred linear model, absent lags, each degree increase in shift-day maximum WBT causes a reduction in productivity of 0.83%. In our binary specification model, a day where maximum WBT exceeds $28\,^{\circ }$C is associated with 5.8% lower productivity compared to a typical day in the estimating sample when it does not.

Note that we observe attendance but not failure to attend a scheduled shift. If we assume that those workers more sensitive to heat are at least more likely to be absent on days when temperature is high than those less sensitive, then our estimates derived from non-absent workers are likely to be *conservative* estimates of population-level effects. We find that shift-level absenteeism rates are higher on hot days, which provides some support to this claim.

Sleep quality is already established to be damaged by high temperatures and humidity. Consistent with this, our estimates of inter-day, night-time, and cumulative heat effects suggest humid heat may impair productivity by undermining the ability of workers to physically recover between shifts. Nevertheless, we estimate substantial same-day effects of heat on productivity even when accounting for these effects.

We caveat our results by pointing out that our data comes from a single activity (wafer slicing) in a single manufacturing facility. We have no reason to suspect that the firm that we study is atypical of the sector, but cannot rule out the possibility that firm-specific factors, for example shift-pattern management practices, choice and maintenance of of machinery, or A/C usage policy, influence how outdoor temperature effects productivity in our setting, such that similar patterns would not be found in other firms or at other facilities. As such it would be valuable in future research to replicate our analysis in other manufacturing sectors and in other locations to get a more complete picture, and to probe external validity more broadly, for example in other sectors that involve different forms of precision manufacturing.

In terms of policy implications, the negative, same-day effect of heat on productivity is important because it is observed in workplaces already benefiting from the protection of good quality climate control technology. Importantly, in terms of understanding the economic burden of high temperatures, the effect sizes should be interpreted as already accounting for the most obvious technological approach to adaptation. Analogous effects will plausibly arise in other high-value precision manufacturing settings.

To the extent that part of our results may be driven by poor recovery between shifts (for example as a result of disturbed sleep), a natural policy recommendation is to expand the coverage of residential air conditioning, especially in parts of the world (such as Southeast Asia) where humid heat is predicted to increase. Our findings provide a cautionary tale to policy-makers that simple adaptive strategies can be expected to protect only partially from the negative consequences of increased heat. The implications for firms are that climate control on its own is not sufficient to insulate the firm in our study from the deleterious effects of high outdoor temperature—and might point to alternative defensive strategies, such as geographical relocation, as potentially valuable in a warming world.

## Figures and Tables

**Fig. 5:**
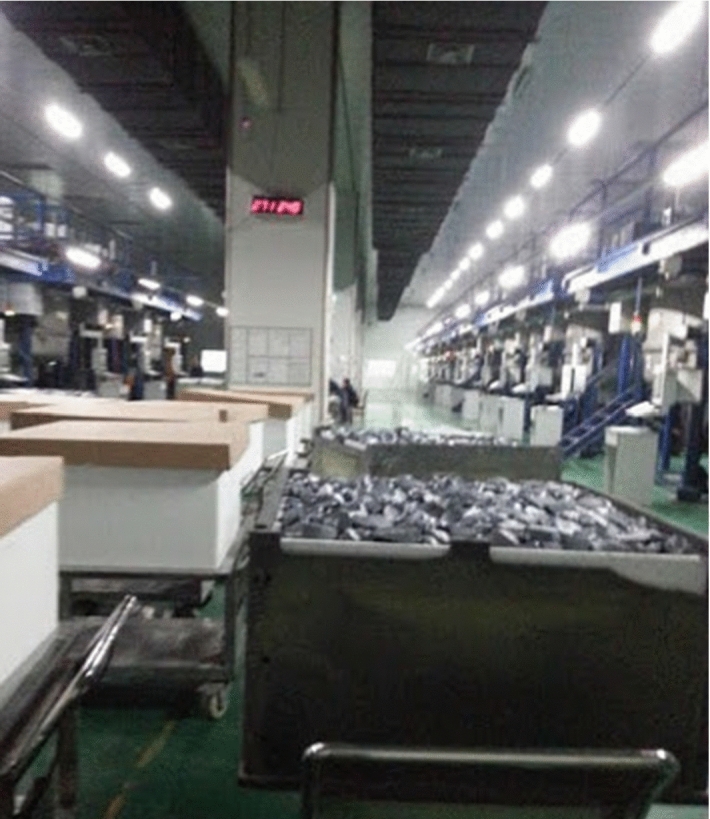
A typical workshop

## A Polynomial Distributed Lag Model

Taking the quadratic polynomial distributed lag model as an example, in order to estimate the parameters of $\beta _{j}$ ($j=0,1,...,s$), we substitute Eqs. (5) to (4) and yield:

7 $$\begin{aligned} f(Temp(t))&= \eta _{0}\sum _{j=0}^{s}Temp_{t-j}+\eta _{1} \sum _{j=0}^{s}j\cdot Temp_{t-j} \nonumber \\&\quad +\eta _{2}\sum _{j=0}^{s}j^{2}\cdot Temp_{t-j}. \end{aligned}$$

By constructing the new regressors of $\sum _{j=0}^{s}Temp_{t-j}$, $\sum _{j=0}^{s}j\cdot Temp_{t-j}$, and $\sum _{j=0}^{s}j^{2}\cdot Temp_{t-j}$, we estimate Eq. (3) with *f*(*Temp*(*t*)) defined by Eq. (7) to get the estimated parameters of $\eta _{0}$, $\eta _{1}$, and $\eta _{2}$. According to Eq. (5), the original model parameters of $\beta _{j}$ ($j=0,1,...,s$) can be derived. For any higher degree of polynomial, a similar procedure can be applied.

The restrictions imposed by a polynomial distributed lag model can be tested as linear restrictions on the parameters of finite distributed lag model. Taking the quadratic polynomial distributed lag model as an example, we difference $\beta _j$ of Eq. (5) in terms of *j* to get

8 $$\begin{aligned} \beta _j-\beta _{j-1}&= \left( \eta _0+\eta _1j+\eta _2j^2\right) -\left( \eta _0+\eta _1(j-1)+\eta _2(j-1)^2 \right) \nonumber \\&=\eta _1+\eta _2(2j-1), \; \; \; j=1,2,...,s, \end{aligned}$$

and then difference Eq. (8) again to get

9 $$\begin{aligned} (\beta _j-\beta _{j-1})-(\beta _{j-1}-\beta _{j-2})&= \left( \eta _1+\eta _2(2j-1)\right) -\left( \eta _1+\eta _2(2(j-1)-1)\right) \nonumber \\&=2\eta _2, \; \; \; j=2,3,...,s. \end{aligned}$$

It shows that the restrictions imposed by quadratic polynomial distributed lag model are equivalent to restricting the second difference of $\beta _j$ of the distributed lag model as a constant, i.e.

10 $$\begin{aligned} \beta _j-2\beta _{j-1}+\beta _{j-2}=\beta _{j-1}-2\beta _{j-2} +\beta _{j-3}, \; \; \; j=3,4,...,s. \end{aligned}$$

Therefore, we can test the restrictions Eq. (10) on the lag coefficients of $\beta _j$ to test the quadratic polynomial lag model.

Following a similar procedure to the fourth difference of $\beta _j$, we can get the restrictions on $\beta _j$ equivalent to those imposed by a quartic polynomial lag model are

11 $$\begin{aligned} \beta _j-4\beta _{j-1}+6\beta _{j-2}-4\beta _{j-3}+\beta _{j-4}=\beta _{j-1}-4 \beta _{j-2}+6\beta _{j-3}-4\beta _{j-4}+\beta _{j-5}, \; \; \; j=5,6,...,s. \end{aligned}$$

## B Sample Characteristics and Additional Results

See Tables

**Table 11:** Variable description

Variable	Description
Output	Total amount of product produced by a worker on a day
Output_A	Total amount of grade-A product produced by a worker on a day
Temperature	Maximum wet bulb temperature of a day
Temperature 24-hr average	Average wet bulb temperature over 24 h of a day
Temperature daytime average	Average wet bulb temperature from 8am to 8pm of a day
Temperature>24	Binary indicator of the maximum wet bulb temperature of a day higher than 24
Temperature>26	Binary indicator of the maximum wet bulb temperature of a day higher than 26
Temperature>28	Binary indicator of the maximum wet bulb temperature of a day higher than 28
Dry bulb temperature	Maximum dry bulb temperature of a day in Celsius
Relative humidity	Maximum relative humidity of a day in per cent
Precipitation	Total precipitation of a day, in millimeter
Cloud coverage	Total cloud coverage, in per cent
Sunshine duration	Sunshine duration over a day, in minute
Wind gust	Average wind gust, in km/hour
Wind speed	Maximum wind speed, in km/hour
PM2_5	in $\upmu \mathrm{{g/m}}^3$
PM10	in $\upmu \mathrm{{g/m}}^3$
SO$_2$	in $\upmu \mathrm{{g/m}}^3$
NO$_2$	in $\upmu \mathrm{{g/m}}^3$
CO	in mg/m$^3$
O$_3$	in $\upmu \mathrm{{g/m}}^3$
Output_d	Daily average amount of product produced by a worker over a week
Output_A_d	Daily average amount of grade-A product produced by a worker over a week
Temp._d	Averaged maximum wet bulb temperature over a week
Temp._hot	Number of days with maximum wet bulb temperature higher than 28 in a week
Precipitation_d	Daily average of total precipitation in a week, in millimeter
Cloud coverage_d	Daily average of total cloud coverage in a week, in per cent
Sunshine duration_d	Daily average of sunshine duration in a week, in minute
Wind gust_d	Average of wind gust in a week, in km/hour
Wind speed_d	Average of maximum wind speed in a week, in km/hour
Age	Worker age in years
Education	Maximum year in education
Slicer	Post of the worker is slicer or not
Lnexp	Ln transformation of worker’s working time (in days) at the factory

11

**Table 12:** Summary statistics for day shift samples

	All		Post Sept. 2013		Apr.–Sept., post Sept. 2013		May–Sept., post Sept. 2013		May–Sept., post Jan. 2015		May–Sept., post Sept. 2013	
	Mean	Std.	Mean	Std.	Mean	Std.	Mean	Std.	Mean	Std.	Mean	Std.
Output	8118.78	4231.70	9441.66	4236.13	9439.05	4077.35	9390.37	3892.88	10196.14	4056.54	9682.81	3932.90
Grade-A output	7037.53	3734.16	8146.67	3776.67	8132.99	3654.21	8083.06	3482.25	8814.16	3634.66	8352.63	3522.31
Temperature	20.23	6.32	20.03	6.14	25.36	3.09	26.15	2.44	26.15	2.48	26.16	2.45
Temperature 24-hr average	18.23	6.16	18.04	6.05	23.44	3.01	24.27	2.34	24.29	2.34	24.30	2.34
Temperature daytime average	18.10	6.07	17.91	5.97	23.23	2.96	24.04	2.31	24.06	2.31	24.06	2.31
Temperature > 24	0.36	0.48	0.34	0.47	0.72	0.45	0.82	0.39	0.82	0.38	0.82	0.39
Temperature >26	0.24	0.42	0.21	0.41	0.48	0.50	0.57	0.49	0.56	0.50	0.57	0.49
Temperature>28	0.10	0.30	0.08	0.27	0.18	0.39	0.22	0.42	0.24	0.43	0.23	0.42
Dry bulb temperature	22.71	8.19	22.52	7.86	28.78	4.18	29.61	3.60	29.60	3.71	29.63	3.63
Relative humidity	86.91	12.73	86.01	13.52	91.62	7.99	92.16	7.54	92.24	7.36	92.15	7.53
Precipitation	3.98	9.58	4.12	9.99	5.62	12.58	5.73	13.21	5.96	14.04	5.74	13.38
Cloud coverage (%)	61.00	36.30	60.40	36.44	60.83	33.35	59.49	33.76	59.74	34.55	59.35	33.96
Sunshine duration	239.72	253.82	240.45	252.29	245.87	241.77	256.94	246.17	257.30	251.23	257.93	247.36
Wind gust	10.18	5.20	10.18	5.02	10.34	5.84	10.02	5.63	10.70	6.04	10.15	5.73
Wind speed	7.99	3.77	7.86	3.73	7.76	3.93	7.52	3.73	7.81	3.92	7.58	3.77
PM2.5									33.23	14.96		
PM10									63.13	23.16		
SO$_2$									25.88	13.18		
NO$_2$									15.59	5.25		
CO									1.32	0.24		
O$_3$									48.22	20.84		
Age											37.19	5.60
Education											11.82	1.84
Slicer											0.40	0.49
Lnexp											7.38	0.94
Observations	141042		98401		42745		35190		24546		29949	
Workers	1854		1048		683		635		437		331	

, 12

**Table 13:** IV estimates (PM2.5, PM10, and SO2)

	(1)	(2)	(3)	(4)	(5)	(6)	(7)	(8)
	OLS	OLS Pref	OLS	IV	OLS	IV	OLS	IV
WBT	$$-$$107.325$$^{***}$$	$$-$$78.001$$^{***}$$	$$-$$80.104$$^{***}$$	$$-$$166.606$$^{***}$$	$$-$$79.307$$^{***}$$	$$-$$154.121$$^{***}$$	$$-$$89.253$$^{***}$$	$$-$$123.465$$^{***}$$
	(13.992)	(9.869)	(12.377)	(33.346)	(12.409)	(29.006)	(12.879)	(19.318)
PM2.5	Yes	No	Yes	Yes	No	No	No	No
PM10	Yes	No	No	No	Yes	Yes	No	No
SO$_2$	Yes	No	No	No	No	No	Yes	Yes
NO$_2$	Yes	No	No	No	No	No	No	No
CO	Yes	No	No	No	No	No	No	No
O$_3$	Yes	No	No	No	No	No	No	No
Obs	24,546	35,190	24,546	24,546	24,546	24,546	24,546	24,546
No. of workers	437	635	437	437	437	437	437	437

and 13.

### B.1 Using Thermal Inversion as Instrumental Variable for Pollution Variables

Following Yao et al. (2022), we construct a measure of thermal inversions by using NASA MERRA dataset. We extract the six-hourly air temperature at the 10kmX10km grid where the factory is located for each of the 72 atmospheric layers, over the sample time period, i.e. from September 2013 to August 2017. To measure thermal inversions, we use the air temperature at the 72nd layer (representing approximately 110 m in altitude) and the 70the layer (representing approximately 550 m in altitude). First, we generate a binary variable indicating thermal inversion occurrence if air temperature at the 70th layer is higher than that at the 72nd layer. Second, we average the four thermal inversion indicators over a day to get a measure of daily thermal inversion. We use this measure as the instrumental variable (IV) for pollution variables. As we only have one IV, but we have six pollution variables, i.e., PM2.5, PM10, SO$_2$, NO$_2$, CO, and O$_3$, we run the IV regression separately for each pollution variable. The results are reported in Table 13 and 14.

See Tables

**Table 14:** IV estimates (NO$_2$, CO, and O$_3$)

	(1)	(2)	(3)	(4)	(5)	(6)	(7)	(8)
	OLS	OLS Pref	OLS	IV	OLS	IV	OLS	IV
WBT	$$-$$107.325$$^{***}$$	$$-$$78.001$$^{***}$$	$$-$$74.811$$^{***}$$	$$-$$102.847$$^{***}$$	$$-$$47.518$$^{***}$$	$$-$$141.765$$^{***}$$	$$-$$106.236$$^{***}$$	$$-$$240.053$$^{***}$$
	(13.992)	(9.869)	(12.217)	(14.575)	(13.226)	(25.141)	(12.621)	(60.652)
PM2.5	Yes	No	No	No	No	No	No	No
PM10	Yes	No	No	No	No	No	No	No
SO$_2$	Yes	No	No	No	No	No	No	No
NO$_2$	Yes	No	Yes	Yes	No	No	No	No
CO	Yes	No	No	No	Yes	Yes	No	No
O$_3$	Yes	No	No	No	No	No	Yes	Yes
Obs	24,546	35,190	24,546	24,546	24,546	24,546	24,546	24,546
No. of workers	437	635	437	437	437	437	437	437

^14^

**Table 15:** Absenteeism

	(1)	(2)
WBT	$-$1.039***	$-$0.971**
	(0.380)	(0.403)
Year FE	Yes	Yes
Month FE	Yes	No
Week FE	No	Yes
Day-of-week FE	Yes	Yes

and 15.

See Fig. 4.

## C Working Environment

Given the delicate nature of the wafer production and for quality control purposes, all workshops are equipped with temperature and humidity control systems. Those systems keep the workshops at a constant temperature of 25 $^{\circ }$C (77F) and a humidity level of 60%. The layout of a typical workshop in the manufacturing plant is shown in Fig. 5.

See Figs. 5, 6,7 and 8.

## Footnotes

1 Each machine cost at the time of purchase in excess of USD 800,000.

2 We unfortunately do not have HR data detailing if shift reallocation was planned or unplanned.

3 For completeness Appendix Table 11 summarises all variables, including those that feature in the robustness exercises but not in the main analysis.

4 Such models are routinely used in many areas of economics, for example to model the effect of advertising expenditure on sales. For examples see Joo et al (2014), Kappe et al (2014).

5 Following Yao et al. (2022), we also consider estimations in which we instrument our pollution controls using thermal inversions. The sign, magnitude and significance of our main variable of interest remain the same. See Table 13 in the Appendix.
